# Detection of ESBL/AmpC-Producing and Fosfomycin-Resistant *Escherichia coli* From Different Sources in Poultry Production in Southern Brazil

**DOI:** 10.3389/fmicb.2020.604544

**Published:** 2021-01-11

**Authors:** Luís Eduardo de Souza Gazal, Leonardo Pinto Medeiros, Miriam Dibo, Erick Kenji Nishio, Vanessa Lumi Koga, Bruna Carolina Gonçalves, Tiela Trapp Grassotti, Taiara Carolaine Leal de Camargo, João Juliano Pinheiro, Eliana Carolina Vespero, Kelly Cristina Tagliari de Brito, Benito Guimarães de Brito, Gerson Nakazato, Renata Katsuko Takayama Kobayashi

**Affiliations:** ^1^Department of Microbiology, Biological Sciences Center, State University of Londrina, Londrina, Brazil; ^2^Postgraduate Program in Animal Health, Avian Health Laboratory, Veterinary Research Institute Desidério Finamor, Agricultural Diagnosis and Research Department, Secretariat of Agriculture Livestock Rural and Development, Eldorado do Sul, Brazil; ^3^Department of Pathology, Clinical Analysis and Toxicology, Health Sciences Center, State University of Londrina, Londrina, Brazil

**Keywords:** Avian, multidrug resistance (MDR), Enterobacteriaceae, fosfomycin, poultry litter, public health

## Abstract

This study discussed the use of antimicrobials in the commercial chicken production system and the possible factors influencing the presence of Extended-spectrum β-lactamase (ESBL)/AmpC producers strains in the broiler production chain. The aim of this study was to perform longitudinal monitoring of ESBL-producing and fosfomycin-resistant *Escherichia coli* from poultry farms in southern Brazil (Paraná and Rio Grande do Sul states) and determine the possible critical points that may be reservoirs for these strains. Samples of poultry litter, cloacal swabs, poultry feed, water, and beetles (*Alphitobius* sp.) were collected during three distinct samplings. Phenotypic and genotypic tests were performed for characterization of antimicrobial resistant strains. A total of 117 strains were isolated and 78 (66%) were positive for ESBL production. The poultry litter presented ESBL positive strains in all three sampled periods, whereas the cloacal swab presented positive strains only from the second period. The poultry litter represents a significant risk factor mainly at the beginning poultry production (odds ratio 6.43, 95% confidence interval 1–41.21, *p* < 0.05). All beetles presented ESBL positive strains. The predominant gene was *bla*_*CTX–M*_ group 2, which occurred in approximately 55% of the ESBL-producing *E. coli*. The *cit* gene was found in approximately 13% of the ESBL-producing *E. coli* as AmpC type determinants. A total of 19 out of 26 fosfomycin-resistant strains showed the *fos*A3 gene, all of which produced ESBL. The correlation between *fos*A3 and *bla*_*CTX–M*_ group 1 (*bla*_*CTX–M55*_) genes was significant among ESBL-producing *E. coli* isolated from Paraná (OR 3.66, 95% CI 1.9–9.68) and these genetic determinants can be transmitted by conjugation to broiler chicken microbiota strains. Our data revealed that poultry litter and beetles were critical points during poultry production and the presence of fosfomycin-resistant strains indicate the possibility of risks associated with the use of this antimicrobial during production. Furthermore, the genetic determinants encoding CTX-M and fosA3 enzymes can be transferred to *E. coli* strains from broiler chicken microbiota, thereby creating a risk to public health.

## Introduction

Antimicrobial resistance is one of the most alarming public health problems in recent years. According to O’Neill (2014), by 2050, bacterial resistance could cause the deaths of approximately 10 million people each year. The widespread use of antimicrobial drugs, both in humans and animals (including livestock animals), has favored the selection and dissemination of bacterial resistance worldwide (World Health Organization, 2014).

Extended-spectrum β-lactamase (ESBL) and AmpC-like enzymes are among the best-known mechanisms of bacterial resistance, which are both mediated by plasmid genes (Ceccarelli et al., 2019) and can be achieved through the horizontal transfer of mobile genetic elements, in both intestinal and extra-intestinal environments (Lazarus et al., 2014). ESBL and AmpC enzymes are capable of hydrolyzing various β-lactam antimicrobials such as cephalosporins and monobactams, increasing the difficulty of treating these infections (Paterson and Bonomo, 2005; Bush and Fisher, 2011).

Initially, the detection of ESBL/AmpC-producing bacteria was related to cases of infection in humans (Smet et al., 2009; Ewers et al., 2012). However, several studies have reported the presence of these resistant strains in animals, whether domestic (Wieler et al., 2011; Bortolami et al., 2019) or livestock (Pitout and Laupland, 2008; Smet et al., 2009; Dierikx et al., 2013; Laube et al., 2013; Dominguez et al., 2018). ESBL/AmpC enzymes are found in Enterobacteriaceae family members, such as *Escherichia coli*, which have often been isolated in livestock, especially during poultry production (Blanc et al., 2006; Carattoli, 2008; Li et al., 2015). Among the most relevant β-lactamases, CTX-M is one of the main enzymes present in *E. coli* that colonize and infect poultry (Olsen et al., 2014).

Worldwide, ESBL-producing Enterobacteriaceae have emerged in farm animals in recent decades (Carattoli, 2008). The pressure exerted by antimicrobial use, particularly in broiler chickens, led to sensitive strains elimination and selection of resistant ones (Saliu et al., 2017). Although antimicrobial resistance is a natural phenomenon, the prevalence of ESBL-producing strains in broilers has increased due to the antimicrobial use in production (Dierikx et al., 2013). Another relevant finding regarding the use of antimicrobials in production is the presence of fosfomycin-resistant strains in poultry carcasses (Cyoia et al., 2019). Fosfomycin is approved in several countries for urinary tract infections treatment in humans (Keating, 2013; Falagas et al., 2019). Thus, the presence of fosfomycin-resistant strains in poultry raises public health safety concerns.

The possibility that bacterial strains, especially ESBL/AmpC-producing and fosfomycin-resistant *E. coli*, may reach the human population via chicken meat consumption is a public health concern because, compared to other types of meat (e.g., pork and beef), chicken meat has been found to become highly contaminated with ESBL-producing bacteria (Friese et al., 2013). Koga et al. (2015) reported the presence of ESBL/AmpC-producing *E. coli* strains isolated from poultry carcasses in southern Brazil. Cyoia et al. (2019) demonstrated that ESBL-producing *E. coli* strains were capable of transferring genes encoding CTX-M enzymes to a human *E. coli* strain. Plasmid incompatibility groups (Inc groups) are categorized by the propagation inability, in the same cell, of two plasmids belonging to the same group. These plasmids may carry resistance genes and can be found in *E. coli* (Datta and Hedges, 1971; Carattoli, 2011). The *bla*CTX-M (e.g., CTX-M15 and CTX-M55) and *bla*TEM genes are associated with plasmids belonging to the IncF and IncI groups (Cantón and Coque, 2006; Carattoli, 2013).

The presence of ESBL/AmpC-producing and fosfomycin-resistant *E. coli* in poultry carcasses reveals that there are possibly critical points during industrial broiler production where these strains can be found and selected by antimicrobials. Thus, the present study aimed to monitor ESBL/AmpC-producing and fosfomycin-resistant *E. coli* in poultry production and determine the possible critical points that may be reservoirs of these strains.

## Materials and Methods

### Farm Characterization

Monitoring was undertaken in the biggest broiler producers states from Brazil. Five farms in the Rio Grande do Sul (RS) state were sampled between February and May 2016, and three farms in the Paraná (PR) state were sampled between January and March 2018. All farms sampled employed an all-in all-out system. In this system, the barns are emptied for slaughter; the poultry litter is turned and covered with a plastic canvas, remaining inside the barn to be reused; the feeders and drinking fountains are disinfected; and the place remains closed for 15 days (depopulation period), until the arrival of new chicks. Sampling was performed in one barn per farm and monitored at three different times: (1) first day (one-day-old restocking chicks), (2) between 20th and 25th days, and (3) between 36th and 38th days of fattening period (Supplementary Table 1). It is important to note that in the first sampling time, chicks samples were collected, but they did not come into contact with water, feed or poultry litter of barn, so that this would not interfere with the analysis of the results. Besides, a questionnaire was given to the producers to obtain information regarding property characteristics, management, and biosecurity.

In the RS state, the production system was characterized as manual. On average, the farmers restocked approximately 15,000 one-day-old chicks in the poultry houses, one or two poultry houses per farm, and the slaughtering period ranged from 38 to 45 days. The water came from artesian wells and was chlorinated in a reservoir present in each poultry house. In general, the poultry litter was composed of rice husk and reused in up to five subsequent flocks without undergoing treatment. The antimicrobials used in the RS farms were enrofloxacin (ENR), halquinol, and virginiamycin.

In the PR state, the production system used was automated (dark house system). The average restocking was approximately 25,000 one-day-old chicks, one or two poultry houses per farm, and the slaughtering period ranged from 38 to 42 days. Similar to the RS farms, the water used in the bird drinkers was chlorinated. The poultry litter was made of wood shavings and reused without undergoing treatment. The antimicrobials used for treatment in the PR farms were norfloxacin (NOR), ciprofloxacin (CIP), and fosfomycin. All barns investigated were disinfected between a previous and a subsequent flock, followed by a depopulation period to approximately 15 days.

### Ethics Statement

The present study was approved by the Animal Ethics Committee of State University of Londrina (CEUA/UEL) (processing number – 22867.2015.23).

### Sampling

The sampling methodology was based on the procedure described by Laube et al. (2013), with some modifications. During each fattening period, samples of poultry litter (boot swab), cloacal swab (20 randomly selected broiler), poultry feed (500 g), and water (500 mL) were collected. In the PR state, samples of *Alphitobius* sp. (approximately 100 beetles), popularly known as “darkling beetle,” were also collected from the farms. The poultry litter was collected using a sterile boot swab by walking the entire house length in a “zigzag” pattern. The poultry feed was obtained directly from the reservoir and water was collected from the farm reservoir taps. Therefore, a total of 45 samples from PR, and 60 samples in RS were collected.

The collected samples were refrigerated (4°C) and sent to the laboratory for processing on the same day. A total of 25 g of feed was weighed and diluted in 225 mL of buffered peptone water, followed by manual homogenization (approximately 10 min). The boot swab was soaked with buffered peptone water, followed by manual homogenization. The water was processed using the multi-tube method (most probable number method), using Lauryl Triptose broth, Brilliant Green broth, and *E. coli* broth (Himedia Laboratories Pvt. Ltd., Mumbai, India). The beetles were processed following the protocol described by Segabinazi et al. (2005). After preprocessing, all samples were plated on MacConkey agar (MC) (Neogen Corporation, Lansing, Michigan, United States) and cefotaxime-supplemented MacConkey agar (MC/CTX) at a concentration of 8 μg/mL to select positive ESBL/AmpC strains (Jarlier et al., 1988).

### *E. coli* Isolation

MacConkey agar and MC/CTX plates were analyzed for the growth of characteristic colonies of *E. coli*. The grew colonies in MC/CTX (possible ESBL/AmpC-producing *E. coli*) were prioritized, with a collection of one to five colonies. In MC/CTX plates that presented no growth, one to five colonies from the MC plates were collected. The colonies were submitted for biochemical identification using media Escola Paulista de Medicina (Probac, Brazil) (Toledo et al., 1982a; Edwards and Ewing, 1986), Motility, Indole and Lysine (Probac, Brazil) (Toledo et al., 1982b; Edwards and Ewing, 1986), and Simmons citrate (Merck, Darmstadt, Germany). *E. coli* positive colonies were stored in Brain Heart Infusion broth (Himedia Laboratories Pvt. Ltd., Mumbai, India) supplemented with 30% glycerol at −20 and −80°C for subsequent phenotypic and genotypic characterization.

### Antimicrobial Susceptibility Test

The antimicrobial susceptibility test was performed using the standard disk diffusion method recommended by the Clinical and Laboratory Standards Institute (CLSI, 2018). The antimicrobials used included several classes, such as β-lactams: cefotaxime (CTX, 30 μg), ceftazidime (CAZ, 30 μg), cefepime (FEP, 30 μg), aztreonam (ATM, 30 μg), cefoxitin (FOX, 30 μg), imipenem (IPM, 30 μg) and amoxicillin-clavulanic acid (AMC, 20/10 μg); quinolones: CIP (5 μg), NOR (10 μg), enrofloxacin (ENR, 10 μg), and nalidixic acid (NAL, 30 μg); sulfonamides: trimethoprim-sulfamethoxazole (SXT, 1.25/23.75 μg); tetracycline (TET, 30 μg); aminoglycosides: gentamicin (GEN, 10 μg), phenicols: chloramphenicol (CHL, 30 μg); nitrofurans: nitrofurantoin (NIT, 300 μg); and fosfomycins: fosfomycin-trometamol (FOT, 200 μg) (Oxoid Ltd., Basingstoke, Hants, United Kingdom). All strains were confirmed for ESBL production using the double-disk approximation test, described by Jarlier et al. (1988). *E. coli* strain ATCC 25922 was used as quality control and results were interpreted based on the CLSI (2018) criteria.

### DNA Template

DNA samples used for polymerase chain reaction (PCR) assays and sequencing, were extracted using Pure Link^®^ Genomic DNA Mini Kit (Invitrogen^®^).

### Detection of ESBL/AmpC Genes in *E. coli* Strains

A previously described PCR method was used for the detection of the following antimicrobial resistance genes: ESBL producer (*bla*_*CTX–M*_ groups – 1, 2, 8, 9, and 25) (Arlet and Philippon, 1991; Woodford et al., 2005); and AmpC-type producer (*mox*, *fox*, *ebc*, *acc*, *dha*, and *cit*) (Pérez-Pérez and Hanson, 2002). The PCR products positive to *bla*_*CTX–M*_ group 1 were characterized for bidirectional Sanger sequencing on ABI-PRISM 3500 XL (Applied Biosystems), following the manufacturer’s recommendations.

### Detection of Other Antimicrobial Resistance Genes

The presence of fosfomycin (*fos*A3) and colistin (*mcr*-1) resistance was examined as described previously by Sato et al. (2013) and Liu et al. (2016), respectively.

### Phylogenetic Analysis

All *E. coli* isolates were assigned to phylogenetic groups A, B1, B2, or D using PCR according to the methodology described by Clermont et al. (2000). Isolates were grouped into the following groups: group A (*chu*A^–^, *yja*A^–^, and TspE4.C2^–^); group B1 (*chu*A^–^, *yja*A^+^, TspE4.C2^–^); group B2 (*chu*A^+^, *yja*A^+^, TspE4.C2- or *chu*A^+^, *yja*A^+^, TspE4.C2^+^); and group D (*chu*A^+^, *yja*A^–^, TspE4.C2^+^).

### Conjugation Experiments

Horizontal transmission of *bla*_*CTX–M*_ and *fos*A3 genes was investigated using conjugation assays. ESBL-producing *E. coli* from poultry litter, harboring the resistance genes, were chosen as donor strains. Non-ESBL-producing *E. coli* from chicks microbiota were selected as possible recipients, based on the antibiogram test and phylogenetic profile. The selected colonies were strains were grown overnight in Luria Bertani (LB) broth (Difco, Sparks, MD, United States) under agitation at 36°C. Then, 1 mL of each strain was centrifuged at 12,000 × *g* for 2 min at 25°C, the supernatants were discarded and resuspended in new LB broth. A total of 100 μL of a recipient strain and 50 μL of a donor strain were added in 3 mL of new LB broth and incubated overnight without shaking at 36°C. Then, 100 μL of the conjugated samples were seeded on LB agar supplemented with GEN (10 μg/mL) and CTX (4 μg/mL), and the plates were incubated at 36°C for growth. The transconjugants selected were used for phylogenetic analysis and tested for the presence of *bla*_*CTX–M*_ and *fos*A3 genes.

### Plasmid-Based Replicon Typing

All isolates were characterized by the Inc group using plasmid-based replicon typing (PBRT; Carattoli et al., 2005). Simplex-PCR was used to recognize the eight incompatibility plasmids: FIA, FIB, FIC, FII, I1, HI1, HI2, and N (Carattoli et al., 2005).

### Enterobacterial Repetitive Intergenic Consensus Sequence–PCR Analysis

A template DNA was used to amplify the repetitive elements from bacterial isolates using the PCR technique to generate DNA fingerprint patterns. The isolates’ clonality was determined by homology among fragments amplified using enterobacterial repetitive intergenic consensus sequence (ERIC)–PCR (Versalovic et al., 1991). Gel analysis was performed using BioNumerics software, version 7.6 (Applied Maths, Sint-Martens-Laten, Belgium). Similarities in the amplicon profile were compared using a DICE coefficient at 1% tolerance and 0.5% optimization. A dendrogram was constructed with the unweighted-pair group method using the arithmetic mean clustering method with a cut-off of 80% similarity (McLellan et al., 2003).

### Statistical Analysis

The data obtained were analyzed using a logistic regression model to calculate the odds ratio (OR) and 95% confidence interval (CI), with a significance level set at *p* < 0.05, and the statistical software R version 3.5.1. In general, the analysis was performed to verify whether there was any significant trend between the isolates producing ESBL and their origin.

## Results

### Poultry Farm Samples

A total of 117 *E. coli* strains were isolated from eight poultry farms in two states, 58 strains from three PR farms, and 59 strains from five RS farms. *E. coli* samples grown in MC/CTX were obtained from poultry litter in all periods in both states, whereas it was only present in cloacal samples isolated from the second period (Table 1). ESBL-producing strains were detected in poultry feed and beetle samples from PR state. In both states, the water samples showed no *E. coli* growth in MC/CTX (Table 1).

**TABLE 1:** Detection of ESBL-producing *E. coli* isolated in MC/CTX agar from farms in PR and RS states.

*E. coli* source	PR farms									RS farms														
	Farm 1			Farm 2			Farm 3			Farm 4			Farm 5			Farm 6			Farm 7			Farm 8		
	1°	2°	3°	1°	2°	3°	1°	2°	3°	1°	2°	3°	1°	2°	3°	1°	2°	3°	1°	2°	3°	1°	2°	3°
Poultry Litter	+	+	+	+	+	+	−	+	+	−	−	+	+	−	+	+	+	−	+	+	+	−	−	+
Poultry	−	+	+	−	+	+	−	+	+	−	+	+	−	+	+	−	+	−	−	+	+	−	+	+
Poultry Feed	−	+	−	−	+	−	−	+	−	−	−	−	−	−	−	−	−	−	−	−	−	−	−	+
Water	−	−	−	−	−	−	−	−	−	−	−	−	−	−	−	−	−	−	−	−	−	−	−	−
Beetle	+	−	+	−	+	+	−	+	+	NS	NS	NS	NS	NS	NS	NS	NS	NS	NS	NS	NS	NS	NS	NS

*(+) Presence; (−) Absence; (NS) Non Sampling.*

**FIGURE 1:**
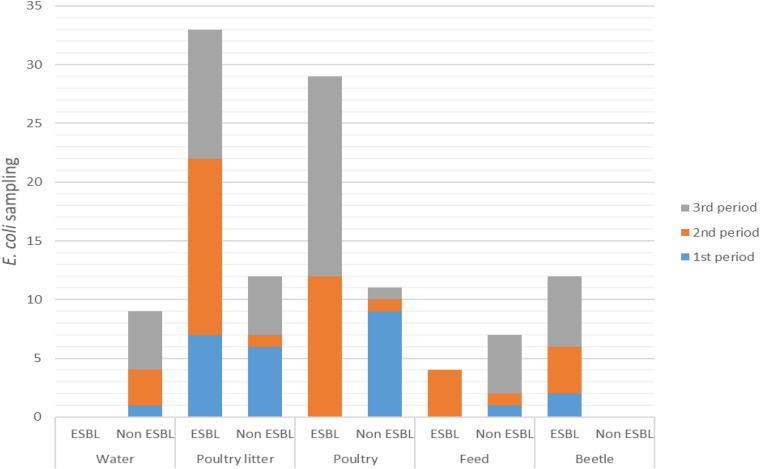
Distribution of ESBL-producing and non-ESBL-producing *E. coli* during the sampling periods.

ESBL-producing *E. coli* were confirmed, respectively, in 49/58 (84%) and 29/59 (49%) isolates from PR and RS. The distribution of ESBL-producing and non-ESBL-producing *E. coli* strains over the sampling periods are shown in Figure 1. Among the 117 samples, ESBL-producing *E. coli* strains were detected in poultry litter in all sampling periods: first period (OR 6.42, 95% CI 1–41.21, *p* < 0.05), second period (OR 3.75, 95% CI 0.4–35.54, *p* < 0.05), and third period (OR 1.05, 95% CI 0.28–3.78, *p* < 0.05). The profile of cloacal poultry strains revealed that ESBL-producing *E. coli* was also present in the second sampling period (OR 2.61, 95% CI 0.27–24.94, *p* < 0.05) and third sampling period (OR 15, 95% CI 1.78–126.59, *p* < 0.05). All the strains isolated from beetles were characterized as ESBL producers from all poultry farms in the PR state (OR > 100, 95% CI 0–inf., *p* < 0.05). No ESBL-producing strains were found in water samples. Moreover, low frequency was found in poultry feed only in the second sampling (OR 0.65, 95% CI 0.06–7.01 *p* < 0.05).

### *E. coli* Antimicrobial Resistance

The antimicrobial susceptibility test indicated that the strains isolated from the poultry farms presented a high frequency of antimicrobial resistance, with 90 and 73% of strains considered as multidrug-resistant (MDR), from PR and RS, respectively (Supplementary Tables 2,3).

In the PR state, most isolates were resistant to CTX (85%), NAL (85%), FEP (81%), TET (79%), SXT (78%), ATM (74%), ENR (66%), CIP, and NOR (55%). Most isolates from the RS state were resistant to GEN (70%), TET (63%), CTX (58%), ATM (56%), NAL (54%), and FEP (51%), presenting a different resistance profile from PR state.

**FIGURE 2:**
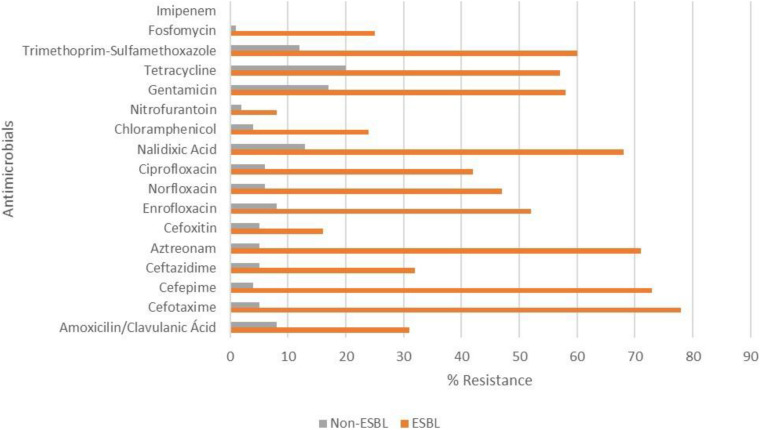
Resistance of ESBL-producing and non-ESBL-producing *E. coli* strains isolated from poultry farms in southern Brazil. There were significant differences between ESBL-producing and non-producing strains (*p* < 0.05), except for cefoxitin, nitrofurantoin and imipenem (all isolates were sensitive).

The frequency of resistance observed for fosfomycin in PR and RS strains was 40 and 5%, respectively. In both states, no IPM-resistant *E. coli* strain was found. ESBL-producing *E. coli* were resistant to a higher number of antimicrobials (except FOX, NIT, and IPM) compared to non-ESBL-producing *E. coli* (*p* < 0.05) (Figure 2).

### Detection of ESBL/AmpC, *fos*A3, and *mcr*-1 Genes

Polymerase chain reaction analysis identified ESBL genes in 92/117 (77%) strains isolated from poultry farms (Supplementary Tables 2,3). Approximately 77/78 (99%) of the ESBL-producing *E. coli* strains were positive for the ESBL genes investigated. In these strains, the genes *bla*_*CTX–M*_ group 2 (55%), *bla*_*CTX–M*_ group 1 (38%), and *bla*_*CTX–M*_ group 8 (10%) were detected. The only AmpC gene detected was *cit*, found in 14/117 isolates, which was associated with *bla*_*CTX–M*_ genes in nine strains. The *bla*_*CTX–M*_ group 9 and *bla*_*CTX–M*_ group 25 genes were not detected and one ESBL-producing strain carried none of the investigated genes. By sequencing, we found that 100% of *bla*_*CTX–M*_ group 1 positive isolates were identified as CTX-M55.

Among the 26 fosfomycin-resistant *E. coli* strains, 19 (73%) harbored the *fos*A3 gene, all had ESBL phenotypes features and 17 of these also harbored the *bla*_*CTX–M55*_ gene (OR 3.66, 95% CI 1.39–9.68, *p* < 0.05). All of these *fos*A3 positive strains were from PR farms that used fosfomycin in broiler production. Three fosfomycin-resistant strains were isolated from the RS state and none of them presented the *fos*A3 gene. The *mcr*-1 gene was detected in only one isolate (EcRS60) from RS. Among the ESBL-producing strains, 14 combinations between the resistance genes detected were observed and most of them are distributed among poultry and poultry litter isolates (Table 2).

**TABLE 2:** Distribution of ESBL-producing *E. coli* strains based on a combination of genes (or CTX-M group genes) and sources in PR and RS states.

	Poultry litter		Poultry		Feed		Beetle	
	PR	RS	PR	RS	PR	RS	PR	RS
CTX-M1	1	–	3	1	1	–	5	NS
CTX-M1 + *fos*A3	6	–	4	–	2	–	3	NS
CTX-M1 + *cit*	–	–	–	1	–	–	–	NS
CTX-M1 + CTX-M2 + CTX-M8	1	–	–	–	–	–	–	NS
CTX-M1 + CTX-M8 + *fos*A3	1	–	–	–	–	–	–	NS
CTX-M1 + *cit* + *fos*A3	–	–	1	–	–	–	–	NS
CTX-M2	7	9	6	10	–	–	4	NS
CTX-M2 + *mcr*-1	–	1	–	–	–	–	–	NS
CTX-M2 + *cit*	–	1	–	–	–	–	–	NS
CTX-M2 + CTX-M8	–	–	–	1	–	–	–	NS
CTX-M2 + CTX-M8 + *cit*	–	2	–	1	–	–	–	NS
CTX-M8 + *cit*	–	–	2	–	–	–	–	NS
*fos*A3	–	–	1	–	1	–	–	NS
*cit*	–	–	–	2–	–	–	–	NS

*(–) Absence; (NS) Non Sampling; Water not presented ESBL-producing *E. coli* positive.*

### Inc Group Plasmid

Tables 2, 3 show the results obtained from the strains submitted to the PBRT technique. Replicons were detected in 97/117 (83%) isolates and the Inc typing showed the presence of FIB (*n* = 89; 76%), I1 (*n* = 37; 32%), HI2 (*n* = 7; 6%), FIA (*n* = 5; 4%), FIC (*n* = 5; 4%), and N (*n* = 4; 3%). The presence of HI1 and FII groups was not detected and 19 strains were negative for all tested replicons. The most frequent replicons among the ESBL-producing samples were FIB (71%) and I1 (36%).

**TABLE 3:** Conjugation experiment between ESBL-producing *E. coli* from poultry litter and non-ESBL-producing *E. coli* from the microbiota of chicks.

	Strain	Phylogenetic group	*bla*_*CTX–M*_ gene	*fos*A3 gene
Donors	EcPR1	B1	*bla*_*CTX–M55*_	+
	EcPR2	B1	*bla*_*CTX–M55*_	+
Recipients	EcRS1	D	*bla*_*CTX–M8*_	−
	EcRS2	D	*bla*_*CTX–M8*_	−
Transconjugants	T1 (EcPR1+EcRS1)	D	*bla*_*CTX–M8 /*_ *bla*_*CTX–M55*_	+
	T4 (EcPR1+EcRS2)	D	*bla*_*CTX–M8 /*_ *bla*_*CTX–M55*_	+

*(+) Presence; (−) Absence.*

### Conjugation Experiments

Two transconjugant strains were obtained in the conjugation assay (T1 and T4), which presented *bla*CTX-M1 and *fos*A3 genes and ESBL phenotype (Table 3).

### Phylogenetic Group and ERIC-PCR Analysis

All strains were assigned to four main phylogenetic groups (Clermont et al., 2000). Most strains belonged to the phylogenetic group D (*n* = 54; 46%), followed by group B1 (*n* = 28; 24%), group A (*n* = 27; 23%), and group B2 (*n* = 8; 7%) (Table 4). The ERIC-PCR analysis showed 16 sub-clusters in the PR state and 15 clusters in the RS state samples, with 19 and 17 singletons remaining, respectively (Supplementary Figures 1,2).

**TABLE 4:** Phylogenetic distribution of 78 ESBL-producing *E. coli* strains and 39 non-ESBL-producing *E. coli* strains based on the isolate source.

	ESBL producer – number of strains (%)					Non-ESBL producer – number of strains (%)					
	A	B1	B2	D	Total	A	B1	B2	D	Total
Poultry Litter	12 (15.4%)	9 (11.5%)	0 (0%)	8 (10.3%)	29	2 (5.1%)	2 (5.1%)	3 (7.7%)	5 (12.8%)	12
Poultry	6 (7.7%)	3 (3.8%)	1 (1.3%)	23 (29.5%)	33	1 (2.6%)	5 (12.8%)	1 (2.6%)	4 (10.3%)	11
Poultry Feed	0 (0%)	1 (1.3%)	0 (0%)	3 (3.8%)	4	4 (10.3%)	0 (0%)	2 (5.1%)	1 (2.6%)	7
Water	0 (0%)	0 (0%)	0 (0%)	0 (0%)	0	2 (5.1%)	4 (10.3%)	0 (0%)	3 (7.7%)	9
Beetle	0 (0%)	4 (5.1%)	1 (1.3%)	7 (9%)	12	NS (0%)	NS (0%)	NS (0%)	NS (0%)	NS

*(NS) Non Sampling.*

## Discussion

The present study revealed a high occurrence of ESBL/AmpC-producing and fosfomycin-resistant *E. coli* in poultry farms from southern Brazil. ESBL phenotype positive strains were found in poultry litter in all sampled periods, and similar was observed by Laube et al. (2013) in Germany. We showed in our study that the chance of ESBL-producing *E. coli* occurrence in poultry litter was significant in all sampling periods, mainly at the beginning of poultry production (OR 6.42). Furthermore, ESBL-producing *E. coli* strains were detected in poultry since the second period, with an increased occurrence in the third period. These data indicate that poultry litter could be a risk factor for ESBL-producing *E. coli* dissemination in poultry houses, including the colonization of one-day-old chicks at the beginning of production. Besides, poultry litter may present a potential risk in the formation of bioaerosols containing antimicrobial-resistant bacteria. Brooks et al. (2010) observed a high concentration of these aerosolized bacteria in poultry houses. The results showed an increase in bacterial concentrations between pre-flock (29%) and late-flock (66%). Studies must be carried out to verify the potential risk of bioaerosols on the health of producers that handle poultry daily. Our results proved that water (OR 0) and poultry feed (OR 0.65) were not sources of ESBL-producing *E. coli* strains.

Besides poultry litter, all *E. coli* strains isolated from beetles were positive for ESBL production (OR > 100). These beetles were collected only in the PR state due their presence and abundance in the local farms. These insects are omnivorous scavengers that feed on fecal matter and debris; therefore, they are commonly found in poultry litter (Axtell, 1999). Many insects that inhabit poultry houses can be carriers or reservoirs of MDR bacteria. Studies performed by Blaak et al. (2014) and Solà-Ginés et al. (2015) showed the presence of ESBL-producing *E. coli* in flies isolated from poultry farms.

Daehre et al. (2018) demonstrated the influence of previous fattening flocks on ESBL-producing strains of the following broiler flock. Through the whole-genome analysis, the authors showed the transmission of ESBL-producing strains between the contaminated environment and poultry, suggesting that cleaning and disinfection practices should be applied. These practices are essential for decreasing the risk of ESBL-producing *E. coli* spreading to the next flocks, according to Mo et al. (2016). The authors analyzed the risk factors for the presence of cephalosporin-resistant *E. coli* during broiler production, which is 0.1 times after a rigorous disinfection process and 9 times after people entering the sheds. These authors suggest that good cleaning practices and the control of people’s access to the barn can reduce the occurrence of these resistant strains in the barn environment. In line with these studies, our work reaffirms the importance of applying disinfection techniques to reduce MDR strains in the breeding broiler environment.

Another biosecurity measure in poultry production is the poultry litter processing by composting, a widely used method to ensure that organic waste is safe before use (Wilkinson et al., 2011). Composting implies a reduction in organic waste volume and considerably decreases pathogenic microorganisms (Bernal et al., 2009). Gazal et al. (2015) analyzed poultry litter after the composting process, observing that few isolates (6.3%) contained virulence genes from extra-intestinal pathogenic *E. coli* and were susceptible to a large number of antimicrobials. Siller et al. (2020) demonstrated that short-term storage reduces the amount of ESBL-producing *E. coli* in poultry litter. Therefore, the practice of composting or storage is a good alternative for eliminating possible pathogens and multiresistant strains.

*Escherichia coli* strains found in water samples were negative for ESBL production. All water from the poultry houses was chlorinated, which excludes the possibility of being a source of ESBL-producing *E. coli*.

The MDR strain frequency in the present study is concerning, and many strains were detected as ESBL-producing. A similar resistance profile has been detected in *E. coli* strains from poultry farms in Italy (Ghodousi et al., 2015) and Germany (Laube et al., 2013). Cyoia et al. (2019) detected MDR in 80% of commercialized chicken carcasses in both PR and RS states. Moreover, 30% of these strains were characterized as ESBL producers. Poultry-derived products are considered the main ESBL-producing bacteria sources among all animal products (Saliu et al., 2017). In Brazil, many antimicrobial agents that have been previously used as growth promoters are now prohibited (MAPA, 2003, 2009); however, some antimicrobials are still used for treatment or as a prophylactic measure. In both states, the investigated farms reported the use of quinolones during poultry production. Moreover, we found high resistance to quinolones, especially in ESBL strains (Figure 2), which also showed high resistance to other antimicrobials, whose use in poultry production was not mentioned, such as GEN, SXT, and TET. Cyoia et al. (2019) also found that ESBL-producing *E. coli* were more resistant to a higher number of antimicrobials than non-ESBL strains. Besides, Zeng and Lin (2017) reported reported the association between the resistance of ESBL and other antimicrobial classes, such as aminoglycosides and fluoroquinolones.

Currently, resistance to third-generation cephalosporins induced by ESBL production represents a major public health problem (Blaak et al., 2015; Centers for Disease Control and Prevention, 2015). These enzymes are very relevant as they confer resistance to antimicrobials used in veterinary medicine, such as penicillin, aminopenicillin, and cephalosporins (e.g., ceftiofur) (Poirel et al., 2018). In the present study, 92/117 (77%) *E. coli* isolates showed *bla* genes (CTX-M1, 2 and 8 groups). Approximately 66% were confirmed as ESBL-producing, and the majority presented the *bla*_*CTX–M*_ group 2 (55%) gene, followed by *bla*_*CTX–M*_ group 1 (38%) and *bla*_*CTX–M*_ group 8 (10%). These data corroborate the description made by Silva and Lincopan (2012) regarding the epidemiology of ESBL genes in Brazilian territory, especially in animal production. Four strains were negative for ESBL phenotype, but harbored both ESBL and AmpC genes (Supplementary Table 2). According to Nishimura et al. (2018), the presence of AmpC determinants interfere with ESBL phenotype and increases false negative detection.

The selective pressure of antimicrobials in poultry farming leads to the death of sensitive strains and also selects the resistant ones (Poole, 2012; Saliu et al., 2017). Moreover, resistance to determined antimicrobials can lead to cross-resistance and co-selection, with stronger promoters increasing ESBL genes’ expression (Gniadkowski, 2008).

Approximately 73% of strains harbored the *fos*A3 gene among the 26 phenotypical fosfomycin-resistant strains. Fosfomycin is a bactericidal antimicrobial that inhibits peptidoglycan synthesis and is widely used in human medicine to treat urinary tract infections (Falagas et al., 2016, 2019). Notably, all the *fos*A3 positive strains were also characterized as ESBL-producing. The relation between *fos*A3 and *bla*_*CTX–M55*_ (*bla*_*CTX–M*_ group 1) genes was significant among ESBL-producing *E. coli* isolated from PR (OR 3.66) and is more evident on strains isolated from RS (OR 6.44, CI 95% 2.89–14.34, *p* < 0.05) since only two strains presented *bla*_*CTX–M55*_ and none strains harboring the *fos*A3 gene were isolated. The *fos*A3 gene was found in isolates from properties that used fosfomycin during poultry production in the PR state. Sato et al. (2013) suggested that ESBL-producing strains that harbor the *bla*_*CTX–M*_ gene (especially the CTX-M55 enzyme) associated with the *fos*A3 gene naturally reside in the intestinal microbiota of individuals in clinical and veterinary environments. Thus, the use of fosfomycin in Brazil’s poultry production may lead to the co-selection of ESBL-producing strains. This is the first study reporting positive strains for the *fos*A3 gene isolated from poultry production in Brazil.

Another important finding was the high heterogeneity among *E. coli* strains analyzed by ERIC-PCR between PR and RS strains that showed several sub-clusters in both states. These results demonstrated that ESBL-producing *E. coli* strains isolated from poultry production in southern Brazil were not caused by clonal spreading. Ghodousi et al. (2015) presented similar data regarding the analysis of ESBL-producing *E. coli* strains isolated from poultry farms in Italy. The spread of ESBL genes among strains isolated from animals occurs by horizontal gene transference (Poirel et al., 2018). Thus, the large number of clusters associated with the high genetic diversity showed in our study suggests that the dissemination of resistance genes is due to horizontal transfer by conjugative plasmids.

IncFIB (*n* = 89; 76%) followed by IncI1 (*N* = 37, 32%) were the dominant replicon types in our samples. Several plasmid families carry ESBL/AmpC genes in ESBL/AmpC-producing Enterobacteriaceae (Carattoli, 2009), of which IncF, IncI, and IncK types are frequently found (Liebana et al., 2012). Our results corroborate with other studies that investigated the presence of ESBL/AmpC-producing *E. coli* strains in poultry (Leverstein-van Hall et al., 2011; Mnif et al., 2012).

The present study also demonstrated the conjugation between ESBL-producing *E. coli* from poultry litter and non-ESBL-producing *E. coli* from chicken microbiota, in which the recipient strains became positive to *bla*_*CTX–M55*_ and *fos*A3 genes (T1 and T4). Cyoia et al. (2019) showed that the genetic determinants encoding CTX-M enzymes were transferred to the J53 strain from a human source. Thus, the conjugation results indicated two relevant points. First, it reinforces the association between *bla*_*CTX–M55*_ and *fos*A3 genes because both were found in the transconjugants. Second, poultry litter could be a starting point for the spreading of resistance genes to poultry and, consequently, to humans via the food chain. Future research intends to clarify the relation between ESBL-producing strains and the production chain in Brazil. In the Netherlands, Apostolakos et al. (2019) showed that ESBL/AmpC producing *E. coli* was prevalent in the broiler production chain, with significant transference to subsequent production levels, indicating that all production levels need to be investigated.

These results show the need for monitoring systems to investigate and understand antimicrobial resistance spreading in animal production, and thus encourage the promotion of antimicrobial rational use. Brazil is the second-largest producer of poultry meat and the leader of exports worldwide (Associação Brasileira de Proteína Animal, 2018). Therefore, Brazil’s production has great relevance in the world poultry scenario and, consequently, influences public health through the food chain.

Currently, the One Health concept has been presented as a way to raise awareness regarding the relation among human, animal, and environmental health (Kahn, 2011). This approach is considered by international organizations, such as the World Health Organization, as an important element in disease control and prevention (Lerner and Berg, 2015). However, this awareness should not only be limited to the control and prevention of pathogens, but also address aspects of antimicrobial resistance. Lammie and Hughes (2016) suggest that due to the challenge presented by antimicrobial resistance and its relation to human, animal, and environmental health, it is essential to include this issue in the One Health approach.

## Conclusion

The antimicrobial use in poultry production has a great influence on MDR bacteria selection and severely influences public health, as well as the national productive sector. A longitudinal monitoring program in poultry production should be implemented to provide data regarding antimicrobial use. The improvement in management techniques, such as poultry litter treatment and good clean practices, may decrease MDR strain frequency in poultry farms. These measures can optimize poultry production and increase animal and human health preservation.

## Data Availability Statement

The raw data supporting the conclusions of this article will be made available by the authors, without undue reservation.

## Ethics Statement

The animal study was reviewed and approved by Animal Ethics Committee of State University of Londrina (CEUA/UEL).

## Author Contributions

LG contributed to the development of experimental research, data analysis, and wrote the manuscript. LM, MD, VK, BG, TG, TC, and JP contributed to the development of experimental research. EN contributed to the statistical analysis. RK, GN, KB, BB, and EV contributed to and assisted in the design of the work and in preparation of the article, and critically reviewed the manuscript. All authors have participated in this study and commented on the manuscript.

## Conflict of Interest

The authors declare that the research was conducted in the absence of any commercial or financial relationships that could be construed as a potential conflict of interest.
