# Supplementary material for: Detection of ESBL/AmpC-Producing and Fosfomycin-Resistant Escherichia coli From Different Sources in Poultry Production in Southern Brazil
Source: Front Microbiol. 2021 Jan 11;11:604544. doi: 10.3389/fmicb.2020.604544 (PMC7829455; doi:10.3389/fmicb.2020.604544)
Supplement: Supplementary file 1 [file Image_1.pdf]

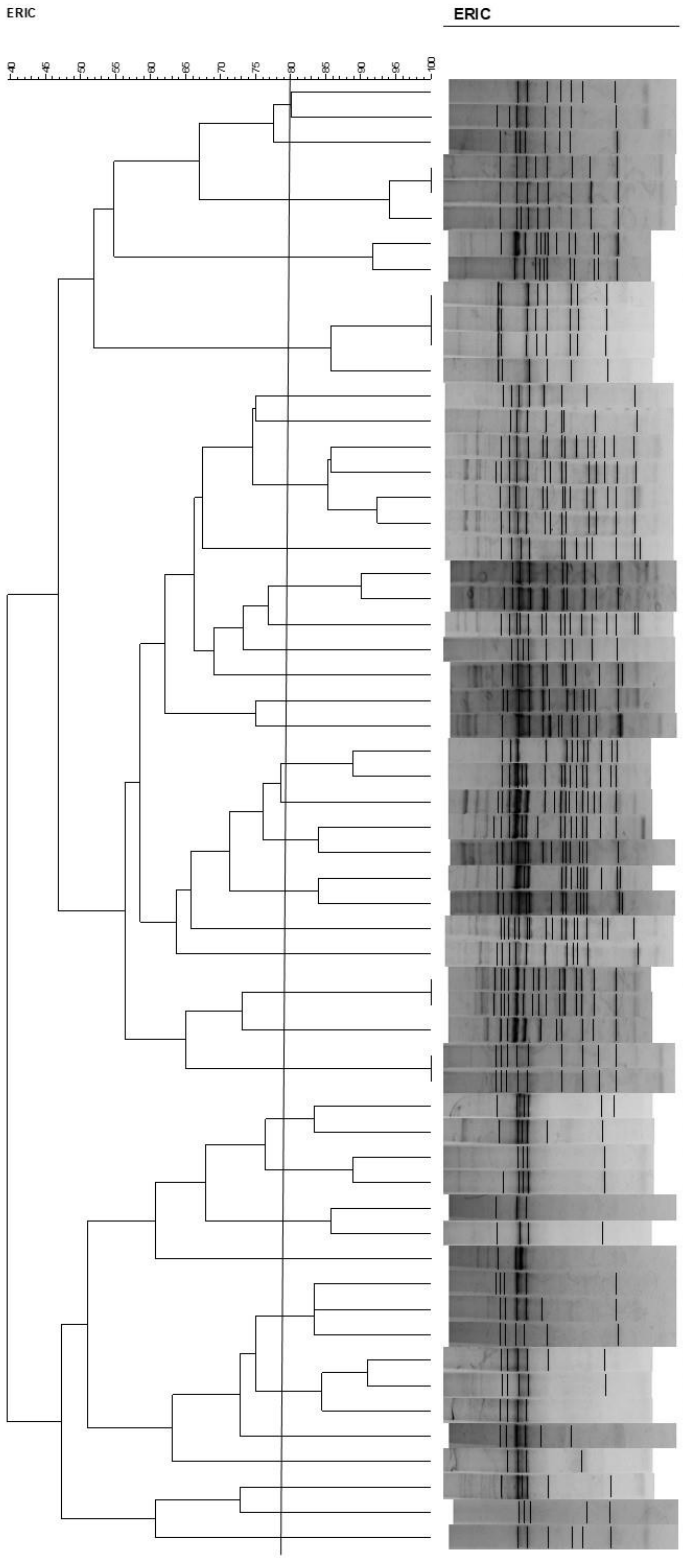

STRAIN	SOURCE	PERIOD	FARM	PG
EcPR34	Poultry litter	1°	3	B2
EcPR35	Poultry litter	1°	3	B2
EcPR41	Poultry	2°	3	D
EcPR31	Poultry	3°	2	D
EcPR57	Poultry	3°	2	D
EcPR30	Poultry	3°	2	D
EcPR5	Beetle	1°	1	D
EcPR6	Beetle	1°	1	D
EcPR51	Beetle	3°	3	D
EcPR52	Beetle	3°	3	D
EcPR53	Beetle	3°	3	D
EcPR45	Poultry litter	3°	3	A
EcPR3	Poultry litter	1°	2	D
EcPR22	Poultry	2°	2	B1
EcPR10	Poultry litter	2°	2	A
EcPR16	Poultry litter	3°	2	A
EcPR12	Beetle	2°	2	B1
EcPR14	Poultry litter	3°	2	A
EcPR4	Poultry litter	1°	2	A
EcPR29	Poultry	3°	1	D
EcPR56	Poultry	3°	1	D
EcPR8	Poultry litter	2°	2	D
EcPR23	Poultry	2°	2	B1
EcPR20	Poultry	2°	1	A
EcPR21	Poultry	2°	1	D
EcPR28	Poultry	3°	1	D
EcPR1	Poultry litter	1°	1	B1
EcPR2	Poultry litter	1°	1	B1
EcPR17	Beetle	3°	1	B2
EcPR15	Poultry litter	3°	1	B1
EcPR24	Water	3°	1	B1
EcPR18	Beetle	3°	1	B1
EcPR25	Water	3°	1	B1
EcPR13	Beetle	2°	2	D
EcPR19	Beetle	3°	2	D
EcPR7	Poultry litter	2°	1	D
EcPR9	Poultry litter	2°	1	D
EcPR11	Feed	2°	1	D
EcPR26	Feed	2°	2	D
EcPR27	Feed	2°	2	D
EcPR44	Beetle	2°	3	B1
EcPR49	Poultry	3°	3	D
EcPR58	Water	3°	3	D
EcPR59	Water	3°	3	D
EcPR43	Poultry litter	2°	3	B1
EcPR54	Beetle	2°	3	B1
EcPR42	Poultry litter	2°	3	B1
EcPR37	Poultry litter	2°	3	B1
EcPR38	Poultry litter	2°	3	D
EcPR39	Poultry	2°	3	D
EcPR48	Poultry	3°	3	D
EcPR55	Poultry litter	3°	3	D
EcPR47	Poultry	3°	3	D
EcPR40	Poultry	2°	3	D
EcPR46	Poultry litter	3°	3	A
EcPR50	Poultry	3°	3	D
EcPR32	Poultry litter	3°	1	B1
EcPR33	Poultry litter	1°	3	B2

Supplementary data Image 1. **ERIC-PCR dendrogram of 58 *E. coli* strains isolated from the PR state poultry farms, with a cut-off of 80% similarity. PG - phylogenetic group.**
