# Supplementary material for: Detection of ESBL/AmpC-Producing and Fosfomycin-Resistant Escherichia coli From Different Sources in Poultry Production in Southern Brazil
Source: Front Microbiol. 2021 Jan 11;11:604544. doi: 10.3389/fmicb.2020.604544 (PMC7829455; doi:10.3389/fmicb.2020.604544)
Supplement: Supplementary file 2 [file Image_2.pdf]

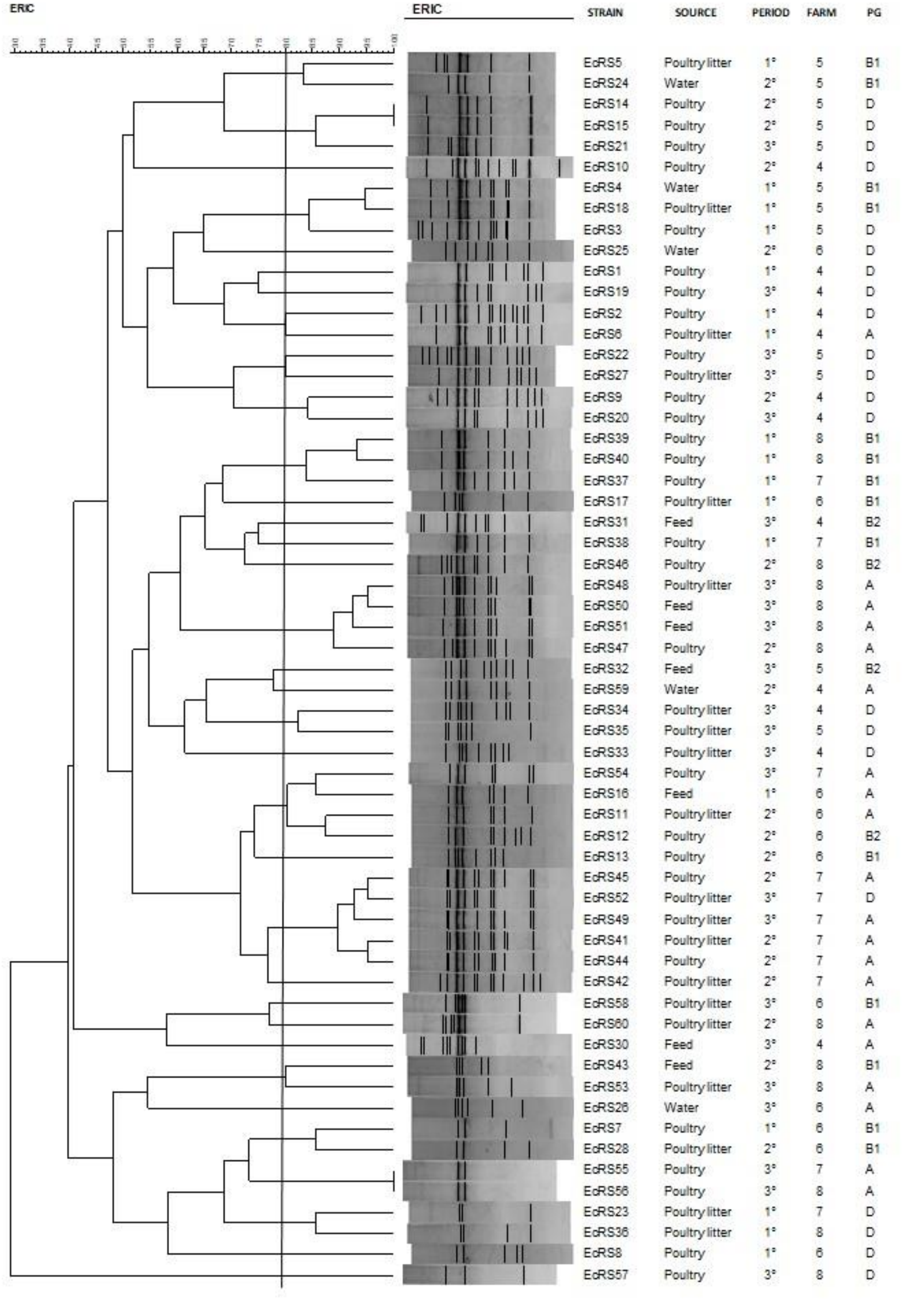

Supplementary data Image 2. **ERIC-PCR dendrogram of 59 *E. coli* strains isolated from the RS state poultry farms, with a cut-off of 80% similarity. PG - phylogenetic group.**
