# Supplementary material for: Detection of ESBL/AmpC-Producing and Fosfomycin-Resistant Escherichia coli From Different Sources in Poultry Production in Southern Brazil
Source: Front Microbiol. 2021 Jan 11;11:604544. doi: 10.3389/fmicb.2020.604544 (PMC7829455; doi:10.3389/fmicb.2020.604544)
Supplement: Supplementary file 3 [file Table_1.doc]

**Supplementary data Table 1**. Description of sampling in the eight broiler farms during February/2016 and May/2018 period.

|  |  |  |  | **N° of Sampling** | | | | |
| --- | --- | --- | --- | --- | --- | --- | --- | --- |
| **Farm** | **Fattering Period** | **Date** | **N° of Barns per Farm** | **Poultry Swab** | **Poultry Litter** | **Water**  **(500 mL)** | **Poultry Feed**  **(500 g)** | **Beetles** |
| **1** | 1° | Jan/18 | 1 | 20 broilers | 2 boot swab | 1 sterile bottle | 1 sterile bag | ± 100 beetles |
|  | 2° | Feb/18 |  | 20 broilers | 2 boot swab | 1 sterile bottle | 1 sterile bag | ± 100 beetles |
|  | 3° | Feb/18 |  | 20 broilers | 2 boot swab | 1 sterile bottle | 1 sterile bag | ± 100 beetles |
| **2** | 1° | Jan/18 | 1 | 20 broilers | 2 boot swab | 1 sterile bottle | 1 sterile bag | ± 100 beetles |
|  | 2° | Feb/18 |  | 20 broilers | 2 boot swab | 1 sterile bottle | 1 sterile bag | ± 100 beetles |
|  | 3° | Feb/18 |  | 20 broilers | 2 boot swab | 1 sterile bottle | 1 sterile bag | ± 100 beetles |
| **3** | 1° | Mar/18 | 1 | 20 broilers | 2 boot swab | 1 sterile bottle | 1 sterile bag | ± 100 beetles |
|  | 2° | Mar/18 |  | 20 broilers | 2 boot swab | 1 sterile bottle | 1 sterile bag | ± 100 beetles |
|  | 3° | Mar/18 |  | 20 broilers | 2 boot swab | 1 sterile bottle | 1 sterile bag | ± 100 beetles |
| **4** | 1° | Feb/16 | 1 | 20 broilers | 2 boot swab | 1 sterile bottle | 1 sterile bag | NS |
|  | 2° | Mar/16 |  | 20 broilers | 2 boot swab | 1 sterile bottle | 1 sterile bag | NS |
|  | 3° | Mar/16 |  | 20 broilers | 2 boot swab | 1 sterile bottle | 1 sterile bag | NS |
| **5** | 1° | Feb/16 | 1 | 20 broilers | 2 boot swab | 1 sterile bottle | 1 sterile bag | NS |
|  | 2° | Mar/16 |  | 20 broilers | 2 boot swab | 1 sterile bottle | 1 sterile bag | NS |
|  | 3° | Mar/16 |  | 20 broilers | 2 boot swab | 1 sterile bottle | 1 sterile bag | NS |
| **6** | 1° | Mar/16 | 1 | 20 broilers | 2 boot swab | 1 sterile bottle | 1 sterile bag | NS |
|  | 2° | Apr/16 |  | 20 broilers | 2 boot swab | 1 sterile bottle | 1 sterile bag | NS |
|  | 3° | Apr/16 |  | 20 broilers | 2 boot swab | 1 sterile bottle | 1 sterile bag | NS |
| **7** | 1° | Apr/16 | 1 | 20 broilers | 2 boot swab | 1 sterile bottle | 1 sterile bag | NS |
|  | 2° | May/16 |  | 20 broilers | 2 boot swab | 1 sterile bottle | 1 sterile bag | NS |
|  | 3° | May/16 |  | 20 broilers | 2 boot swab | 1 sterile bottle | 1 sterile bag | NS |
| **8** | 1° | Apr/16 | 1 | 20 broilers | 2 boot swab | 1 sterile bottle | 1 sterile bag | NS |
|  | 2° | May/16 |  | 20 broilers | 2 boot swab | 1 sterile bottle | 1 sterile bag | NS |
|  | 3° | May/16 |  | 20 broilers | 2 boot swab | 1 sterile bottle | 1 sterile bag | NS |

NS – Non sampling
