# Supplementary material for: Detection of ESBL/AmpC-Producing and Fosfomycin-Resistant Escherichia coli From Different Sources in Poultry Production in Southern Brazil
Source: Front Microbiol. 2021 Jan 11;11:604544. doi: 10.3389/fmicb.2020.604544 (PMC7829455; doi:10.3389/fmicb.2020.604544)
Supplement: Supplementary file 4 [file Table_2.doc]

Supplementary data Table 2. **Phenotypic and genotypic characteristics of *E. coli* strains isolated from poultry farms in Paraná.**

| **Strain** | **Source** | **Farm** | **Period** | **PG*** | **Antimicrobial Resistance** | **Phenotypic ESBL** | **Inc. Group** | **ESBL/AmpC genes** | **mcr-1 gene** | ***fosA3* gene** |
| --- | --- | --- | --- | --- | --- | --- | --- | --- | --- | --- |
| **EcPR01** | Poultry litter | 1 | 1° | B1 | ATM – FEP – CTX – ENR – NOR – CIP – CHL – SXT – FOT – TET – NAL | + | - | *bla*_CTX-M 1_ | - | + |
| **EcPR02** | Poultry litter | 1 | 1° | B1 | ATM – CAZ – FEP – CTX – ENR – NOR – CIP – CHL – NIT – SXT – FOT – TET – NAL | + | - | *bla*_CTX-M 1_ | - | + |
| **EcPR03** | Poultry litter | 2 | 1° | D | AMC – ATM – FEP – CTX – ENR – NOR – CIP – GEN – NAL | + | - | *bla*_CTX-M 2_ | - | - |
| **EcPR04** | Poultry litter | 2 | 1° | A | AMC – ATM – FEP – CTX – ENR – CHL – SXT – GEN – NAL | + | FIB | *bla*_CTX-M 2_ | - | - |
| **EcPR05** | Beetle | 1 | 1° | D | ATM – FEP – CTX – ENR – NOR – CIP – CHL – SXT – FOT – TET – GEN – NAL | + | - | *bla*_CTX-M 1_ | - | + |
| **EcPR06** | Beetle | 1 | 1° | D | ATM – FEP – CTX – ENR – NOR – CIP – CHL – SXT – FOT – TET – GEN – NAL | + | - | *bla*_CTX-M 1_ | - | + |
| **EcPR07** | Poultry litter | 1 | 2° | D | AMC – ATM – FEP – CTX – ENR – NOR – CIP – CHL – SXT – TET – NAL | + | FIB | *bla*_CTX-M 2_ | - | - |
| **EcPR08** | Poultry litter | 2 | 2° | D | ATM – FEP – CTX – ENR – NOR – CIP – CHL – SXT – TET – NAL | + | FIB | *bla*_CTX-M 1_ / *bla*_CTX-M 2_ /*bla*_CTX-M 8_ | - | - |
| **EcPR09** | Poultry litter | 1 | 2° | D | ATM – FEP – CTX – ENR – NOR – CIP – CHL – SXT – TET – NAL – FOX | + | FIB | *bla*_CTX-M 2_ | - | - |
| **EcPR10** | Poultry litter | 2 | 2° | A | FEP – CTX – ENR – FOT – TET – NAL | + | FIB - FIC | *bla*_CTX-M 1_ | - | + |
| **EcPR11** | Feed | 1 | 2° | D | ATM – CAZ – FEP – CTX – ENR – NOR – CIP – CHL – NIT – SXT – FOT – TET – GEN – NAL | + | I1 | *bla*_CTX-M 1_ | - | + |
| **EcPR12** | Beetle | 2 | 2° | B1 | ATM – CAZ – FEP – CTX – ENR – NOR – CIP – CHL – SXT – TET – GEN – NAL | + | I1 | *bla*_CTX-M 1_ | - | - |
| **EcPR13** | Beetle | 2 | 2° | D | ATM – FEP – CTX – ENR – SXT – TET – NAL | + | I1 – H2 – FIB | *bla*_CTX-M 1_ | - | - |
| **EcPR14** | Poultry litter | 2 | 3° | A | ATM – CAZ – FEP – CTX – ENR – NOR – CIP – CHL – FOT – TET – NAL | + | FIB – N | *bla*_CTX-M 1_ | - | + |
| **EcPR15** | Poultry litter | 1 | 3° | B1 | ATM – FEP – CTX – ENR – NOR – CIP – CHL – SXT – FOT – TET – NAL | + | FIB – N | *bla*_CTX-M 1_ /*bla*_CTX-M 8_ | - | + |
| **EcPR16** | Poultry litter | 2 | 3° | A | ATM – CAZ – FEP – CTX – ENR – NOR – CIP – CHL – SXT – FOT – TET – NAL | + | FIB – N | *bla*_CTX-M 1_ | - | + |
| **EcPR17** | Beetle | 1 | 3° | B2 | AMC – ATM – CAZ – FEP – CTX – ENR – NOR – CIP – CHL – NIT – TET – NAL | + | I1 - FIB | *bla*_CTX-M 2_ | - | - |
| **EcPR18** | Beetle | 1 | 3° | B1 | AMC – ATM – FEP – CTX – CHL – FOT – TET – NAL | + | FIB | *bla*_CTX-M 1_ | - | + |
| **EcPR19** | Beetle | 2 | 3° | D | AMC – ATM – FEP – CTX – ENR – NOR – CIP – CHL – SXT – FOT – TET – NAL | + | I1 - FIB | *bla*_CTX-M 1_ | - | - |
| **EcPR20** | Poultry | 1 | 2° | A | AMC – ATM – CAZ – FEP – CTX – ENR – NOR – CIP – CHL – SXT – FOT – TET – NAL | + | N | *bla*_CTX-M 1_ | - | - |
| **EcPR21** | Poultry | 1 | 2° | D | AMC – ATM – CAZ – FEP – CTX – ENR – NOR – CIP – CHL – SXT – FOT – TET – GEN – NAL – FOX | + | FIA – FIB | *bla*_CTX-M 1_ / *cit* | - | + |
| **EcPR22** | Poultry | 2 | 2° | B1 | AMC – ATM – CAZ – FEP – CTX – ENR – NOR – CIP – CHL – NIT – SXT – FOT – TET – GEN – NAL | + | - | *bla*_CTX-M 1_ | - | - |
| **EcPR23** | Poultry | 2 | 2° | B1 | ATM – CTX – ENR – NOR – CIP – SXT – FOT – TET – NAL | + | I1 | *bla*_CTX-M 1_ | - | + |
| **EcPR24**** | Water | 1 | 3° | B1 | NAL | - | FIB | - | - | - |
| **EcPR25**** | Water | 1 | 3° | B1 | NAL | - | I1 - FIB | - | - | - |
| **EcPR26** | Feed | 2 | 2° | D | AMC – ATM – CAZ – FEP – CTX – ENR – NOR – CIP – CHL – SXT – FOT – TET – NAL | + | - | *-* | - | + |
| **EcPR27** | Feed | 2 | 2° | D | ATM – FEP – CTX – ENR – NOR – CIP – CHL – SXT – FOT – TET – GEN – NAL | + | I1 | *bla*_CTX-M 1_ | - | + |
| **EcPR28** | Poultry | 1 | 3° | D | CTX – ENR – NOR – CIP – FOT – TET – NAL | + | - | *bla*_CTX-M 1_ | - | + |
| **EcPR29** | Poultry | 1 | 3° | D | AMC – ATM – CAZ – FEP – CTX – ENR – NOR – CIP – CHL – FOT – TET – GEN – NAL | + | I1 – FIB |  | - | + |
| **EcPR30** | Poultry | 2 | 3° | D | ATM – FEP – CTX – ENR – NOR – CIP – NIT – SXT – FOT – TET – GEN – NAL | + | - | *bla*_CTX-M 1_ | - | + |
| **EcPR31** | Poultry | 2 | 3° | D | ATM – FEP – CTX – ENR – NOR – CIP – NIT – SXT – FOT – TET – GEN – NAL | + | N | *bla*_CTX-M 1_ | - | - |
| **EcPR32** | Poultry litter | 1 | 3° | B1 | ATM – FEP – CTX – ENR – NOR – CIP – CHL - FOT – SXT - TET – GEN– NAL | + | FIB | *bla*_CTX-M 1_ | - | + |
| **EcPR33**** | Poultry litter | 3 | 1° | B2 | SXT – GEN | *-* | FIB | *bla*_CTX-M 8_ | - | - |
| **EcPR34**** | Poultry litter | 3 | 1° | B2 | SXT – GEN | *-* | FIB | *bla*_CTX-M 8_ | - | - |
| **EcPR35**** | Poultry litter | 3 | 1° | B2 | SXT – GEN | *-* | FIB | *bla*_CTX-M 8_ | - | - |
| **EcPR37** | Poultry litter | 3 | 2° | B1 | FEP – CTX – NIT – SXT – TET – GEN – NAL | + | I1 – HI2 – FIB | *bla*_CTX-M 2_ | - | - |
| **EcPR38**** | Poultry litter | 3 | 2° | D | ENR – NOR – CIP – SXT – TET – GEN – NAL | - | FIA – FIB | - | - | - |
| **EcPR39** | Poultry | 3 | 2° | D | AMC – CAZ – FEP – CTX – SXT – TET – GEN – FOX | + | FIA – FIB | *bla*_CTX-M 8_ / *cit* | - | - |
| **EcPR40** | Poultry | 3 | 2° | D | ATM – FEP – CTX – ENR – NOR – CIP – SXT – TET – GEN – NAL | + | FIA – FIB | *bla*_CTX-M 2_ | - | - |
| **EcPR41** | Poultry | 3 | 2° | D | ATM – FEP – CTX – TET – GEN – NAL | + | FIB | *bla*_CTX-M 2_ | - | - |
| **EcPR42** | Poultry litter | 3 | 2° | B1 | ATM – CAZ – FEP – CTX – SXT – TET – GEN | + | FIB | *bla*_CTX-M 1_ | - | - |
| **EcPR43** | Feed | 3 | 2° | B1 | ATM – FEP – CTX – SXT – TET – GEN | + | FIB | *bla*_CTX-M 1_ | - | - |
| **EcPR44** | Beetle | 3 | 2° | B1 | FEP – CTX – SXT – TET – GEN | + | FIB | *bla*_CTX-M 1_ | - | - |
| **EcPR45** | Poultry litter | 3 | 3° | A | ATM – FEP – CTX – SXT – TET – GEN – NAL | + | FIB | *bla*_CTX-M 2_ | - | - |
| **EcPR46**** | Poultry litter | 3 | 3° | A | GEN - NAL | *-* | - | *bla*_CTX-M 2_ | - | - |
| **EcPR47** | Poultry | 3 | 3° | D | FEP – CTX – ENR – NOR – CIP – SXT – GEN – NAL | + | I1 | *bla*_CTX-M 2_ | - | - |
| **EcPR48** | Poultry | 3 | 3° | D | ATM – FEP – CTX – ENR – CHL – SXT – NAL | + | I1 | *bla*_CTX-M 2_ | - | - |
| **EcPR49** | Poultry | 3 | 3° | D | ATM – FEP – CTX – ENR – CIP – SXT – TET – GEN – NAL | + | FIB | *bla*_CTX-M 2_ | - | - |
| **EcPR50** | Poultry | 3 | 3° | D | AMC – ATM – CAZ – FEP – CTX – NOR – SXT – TET – GEN – NAL – FOX | + | FIB | *bla*_CTX-M 8_ /*cit* | - | - |
| **EcPR51** | Beetle | 3 | 3° | D | ATM – FEP – CTX – SXT – TET – GEN – NAL | + | I1 - FIB | *bla*_CTX-M 2_ | - | - |
| **EcPR52** | Beetle | 3 | 3° | D | ATM – FEP – CTX – SXT – TET – GEN – NAL | + | I1 - FIB | *bla*_CTX-M 2_ | - | - |
| **EcPR53** | Beetle | 3 | 3° | D | ATM – FEP – CTX – SXT – TET – GEN – NAL | + | I1 - FIB | *bla*_CTX-M 2_ | - | - |
| **EcPR54** | Beetle | 3 | 2° | B1 | ATM – FEP – CTX – SXT – TET – GEN | + | I1 - FIB | *bla*_CTX-M 1_ | - | - |
| **EcPR55** | Poultry litter | 3 | 3° | D | ATM – FEP – CTX – TET – GEN – NAL | + | FIB | *bla*_CTX-M 2_ | - | - |
| **EcPR56** | Poultry | 1 | 3° | D | ATM – FEP – CTX – ENR – NOR – CIP – TET – GEN – NAL | + | FIB | *bla*_CTX-M 2_ | - | - |
| **EcPR57** | Poultry | 2 | 3° | D | ATM – CAZ – FEP – CTX – ENR – NOR – CIP – NIT – SXT – FOT – TET – GEN – NAL | + | - | *bla*_CTX-M 1_ | - | + |
| **EcPR58**** | Water | 3 | 3° | D | ENR – SXT – GEN – NAL | - | - | - | - | - |
| **EcPR59**** | Water | 3 | 3° | D | ENR – SXT – GEN | - | - | - | - | - |

(+) Presence; (-) Absence**. *PG**: phylogenetic group; *******E. coli* strains isolated in MacConkey agar without supplement with cefotaxime;
