# Supplementary material for: Detection of ESBL/AmpC-Producing and Fosfomycin-Resistant Escherichia coli From Different Sources in Poultry Production in Southern Brazil
Source: Front Microbiol. 2021 Jan 11;11:604544. doi: 10.3389/fmicb.2020.604544 (PMC7829455; doi:10.3389/fmicb.2020.604544)
Supplement: Supplementary file 5 [file Table_3.doc]

**Supplementary data Table 3. Phenotypic and genotypic characteristics of *E. coli* strains isolated from poultry farms in Rio Grande do Sul.**

| **Strain** | **Source** | **Farm** | **Period** | **PG*** | **Antimicrobial Resistance** | **Phenotypic ESBL** | **Inc. Group.** | **ESBL/AmpC genes** | ***mcr-1* gene** | ***fosA3* gene** |
| --- | --- | --- | --- | --- | --- | --- | --- | --- | --- | --- |
| **EcRS01**** | Poultry | 4 | 1° | D | TET – GEN | *-* | FIB | *bla*_CTX-M 8_ | - | - |
| **EcRS02**** | Poultry | 4 | 1° | D | AMC – TET – GEN – NAL | *-* | FIB | *bla*_CTX-M 8_ | - | - |
| **EcRS03**** | Poultry | 5 | 1° | D | AMC – TET – GEN | *-* | FIB | *-* | - | - |
| **EcRS04**** | Water | 5 | 1° | B1 | - | *-* | FIA - FIB – FIC | - | - | - |
| **EcRS05**** | Poultry litter | 5 | 1° | B1 | TET – GEN | *-* | FIB - FIC | *-* | - | - |
| **EcRS06**** | Poultry litter | 4 | 1° | A | AMC – CHL – TET – NAL | - | FIB | - | - | - |
| **EcRS07**** | Poultry | 6 | 1° | B1 | - | *-* | I1 - FIB | *-* | - | - |
| **EcRS08**** | Poultry | 6 | 1° | D | - | *-* | I1 - FIB | *-* | - | - |
| **EcRS09** | Poultry | 4 | 2° | D | AMC – ATM – FEP – CTX – ENR – NOR – CIP – SXT – TET – GEN – NAL | *+* | I1 - FIB | *bla*_CTX-M 2_ | - | - |
| **EcRS10** | Poultry | 4 | 2° | D | AMC – ATM – CAZ – FEP – CTX – ENR – NOR – CIP – TET – GEN – NAL – FOX | *+* | I1 - FIB | *bla*_CTX-M 2_ | - | - |
| **EcRS11** | Poultry litter | 6 | 2° | A | AMC – ATM – CAZ – FEP – CTX – SXT – GEN – NAL | *+* | FIB | *bla*_CTX-M 2_ | - | - |
| **EcRS12** | Poultry | 6 | 2° | B2 | AMC – ATM – CAZ – FEP – CTX – SXT – GEN – NAL – FOX | *+* | I1 - FIB | *bla*_CTX-M 2_ / *bla*_CTX-M 8_ | - | - |
| **EcRS13** | Poultry | 6 | 2° | B1 | AMC – ATM – CAZ – CTX – ENR – NOR – CIP – SXT – TET – GEN – NAL – FOX | *+* | FIB | *cit* | - | - |
| **EcRS14** | Poultry | 5 | 2° | D | AMC – ATM – CAZ – FEP – CTX – ENR – NOR – CIP – TET – GEN – NAL | *+* | FIB | *bla*_CTX-M 1_ | - | - |
| **EcRS15** | Poultry | 5 | 2° | D | AMC – ATM – CAZ – FEP – CTX – ENR – NOR – SXT – TET – GEN – NAL – FOX | *+* | I1 – FIB | *bla*_CTX-M 2_ | - | - |
| **EcRS16**** | Feed | 6 | 1° | A | NAL | *-* | FIB | - | - | - |
| **EcRS17** | Poultry litter | 6 | 1° | B1 | ATM – FEP – CTX – FOT – TET – GEN | *+* | I1 – HI2 – FIB | *bla*_CTX-M 2_ | - | - |
| **EcRS18** | Poultry litter | 5 | 1° | B1 | AMC – ATM – CAZ – FEP – CTX – ENR – NOR – CIP – CHL – TET – GEN – NAL – FOX | *+* | FIB | *bla*_CTX-M 2_ | - | - |
| **EcRS19** | Poultry | 4 | 3° | D | AMC – CAZ – CTX – ENR – NOR – CIP – SXT – GEN – NAL – FOX | *+* | I1 – FIB | *bla*_CTX-M 1_ / *cit* | - | - |
| **EcRS20** | Poultry | 4 | 3° | D | AMC – ATM – FEP – CTX – ENR – NOR – CIP – SXT – TET – GEN – NAL | *+* | FIB | *bla*_CTX-M 2_ | - | - |
| **EcRS21** | Poultry | 5 | 3° | D | AMC – ATM – CAZ – FEP – CTX – ENR – NOR – TET – GEN – NAL | *+* | FIB | *bla*_CTX-M 2_ | - | - |
| **EcRS22** | Poultry | 5 | 3° | D | AMC – ATM – CAZ – FEP – CTX – ENR – NOR – CIP – TET – GEN – NAL | *+* | I1 – FIB | *bla*_CTX-M 2_ | - | - |
| **EcRS23** | Poultry litter | 7 | 1° | D | AMC – ATM – CAZ – FEP – CTX – FOT – TET – GEN – FOX | *-* ^a^ | HI2 | *bla*_CTX-M 2_ /*cit* | - | - |
| **EcRS24**** | Water | 5 | 2° | B1 | - | - | I1 – FIB – FIC | - | - | - |
| **EcRS25**** | Water | 6 | 2° | D | - | - | FIB | - | - | - |
| **EcRS26**** | Water | 6 | 3° | A | - | *-* | I1 – FIB – FIC | *-* | - | - |
| **EcRS27** | Poultry litter | 5 | 3° | D | AMC – ATM – CAZ – FEP – CTX – ENR – NOR – CIP – TET – GEN – NAL | *+* | I1 – FIB | *bla*_CTX-M 2_ | - | - |
| **EcRS28** | Poultry litter | 6 | 2° | B1 | ATM – FEP – CTX – ENR – NOR – CIP – SXT – TET – GEN – NAL | *+* | HI2 – FIB | *bla*_CTX-M 2_ | - | - |
| **EcRS30**** | Feed | 4 | 3° | A | TET | *-* | FIB | *-* | - | - |
| **EcRS31**** | Feed | 4 | 3° | B2 | TET – FOX | *-* | FIB | *-* | - | - |
| **EcRS32**** | Feed | 5 | 3° | B2 | - | - | I1 – FIB | - | - | - |
| **EcRS33** | Poultry litter | 4 | 3° | D | TET | - | FIB | - | - | - |
| **EcRS34** | Poultry litter | 4 | 3° | D | ENR – NOR – CIP – NIT – TET – NAL | *-* | FIB | *-* | - | - |
| **EcRS35** | Poultry litter | 5 | 3° | D | ENR – NOR – CIP – NIT – TET – NAL | *-* | FIB | *-* | - | - |
| **EcRS36** | Poultry litter | 8 | 1° | D | AMC – ATM – CAZ – FEP – CTX – FOT – TET – GEN – FOX | *+* | I1 – HI2 – FIB | *bla*_CTX-M 2_ /*cit* | - | - |
| **EcRS37**** | Poultry | 7 | 1° | B1 | CHL – TET – GEN | *-* | FIB | *bla*_CTX-M 8_ | - | - |
| **EcRS38**** | Poultry | 7 | 1° | B1 | CHL – SXT – TET – GEN | *-* | HI2 – FIB | - | - | - |
| **EcRS39**** | Poultry | 8 | 1° | B1 | - | *-* | FIB | *bla*_CTX-M 8_ | - | - |
| **EcRS40**** | Poultry | 8 | 1° | B1 | - | *-* | I1 – FIB | *bla*_CTX-M 8_ | - | - |
| **EcRS41** | Poultry litter | 7 | 2° | A | ATM – FEP – CTX – SXT – GEN – NAL | *+* | I1 – FIB | *bla*_CTX-M 2_ | - | - |
| **EcRS42** | Poultry litter | 7 | 2° | A | ATM – CTX – ENR – SXT – GEN – NAL | *+* | I1 | *bla*_CTX-M 2_ | - | - |
| **EcRS43**** | Feed | 8 | 2° | B1 | FEP – TET | - | I1 – FIB | - | - | - |
| **EcRS44** | Poultry | 7 | 2° | A | ATM – FEP – CTX – ENR – SXT – GEN – NAL – FOX | *+* | I1 – FIB | *bla*_CTX-M 2_ | - | - |
| **EcRS45** | Poultry | 7 | 2° | A | ATM – FEP – CTX – SXT – GEN – NAL | *+* | FIB | *bla*_CTX-M 2_ | - | - |
| **EcRS46** | Poultry | 8 | 2° | B2 | AMC – ATM – CAZ | *-* | FIB | *cit* | - | - |
| **EcRS47** | Poultry | 8 | 2° | A | AMC – ATM – CAZ – FEP – CTX – SXT – TET – GEN – FOX | *+* | FIB | *bla*_CTX-M 2_ / *bla*_CTX-M 8_ / *cit* | - | - |
| **EcRS48** | Poultry litter | 8 | 3° | A | AMC – ATM – CAZ – FEP – CTX – SXT – TET – GEN – FOX | *+* | FIB | *bla*_CTX-M 2_ / *bla*_CTX-M 8_ / *cit* | - | - |
| **EcRS49** | Poultry litter | 7 | 3° | A | ATM – FEP – CTX – ENR – SXT – GEN – NAL | *+* | - | *bla*_CTX-M 2_ | - | - |
| **EcRS50** | Feed | 8 | 3° | A | AMC – ATM – CAZ – FEP – CTX – SXT – TET – GEN – FOX | *-* ^a^ | FIB | *bla*_CTX-M 2_ / *bla*_CTX-M 8_ / *cit* | - | - |
| **EcRS51** | Feed | 8 | 3° | A | AMC – ATM – CAZ – FEP – CTX – SXT – TET – GEN – FOX | *-* ^a^ | FIB | *bla*_CTX-M 2_ /*bla*_CTX-M 8_ / *cit* | - | - |
| **EcRS52** | Poultry litter | 7 | 3° | D | ATM – FEP – CTX – SXT – GEN – NAL | *+* | - | *bla*_CTX-M 2_ | - | - |
| **EcRS53** | Poultry litter | 8 | 3° | A | AMC – ATM – CAZ – FEP – CTX – SXT – TET – GEN – FOX | *+* | I1 – FIB | *bla*_CTX-M 2_ / *bla*_CTX-M 8_ / *cit* | - | - |
| **EcRS54** | Poultry | 7 | 3° | A | ATM – CAZ – FEP – CTX – ENR – SXT – GEN – NAL | *+* | - | *bla*_CTX-M 2_ | - | - |
| **EcRS55** | Poultry | 7 | 3° | A | ATM – FEP – CTX – ENR – SXT – GEN – NAL | *+* | - | *bla*_CTX-M 2_ | - | - |
| **EcRS56** | Poultry | 8 | 3° | A | AMC – ATM – CAZ – FEP – CTX – SXT – TET – GEN – FOX | *-* ^a^ | FIB | *bla*_CTX-M 2_ /*bla*_CTX-M 8_ / *cit* | - | - |
| **EcRS57** | Poultry | 8 | 3° | D | AMC – ATM – CAZ – CTX – ENR – NOR – CIP – SXT – TET – GEN – NAL – FOX | *+* | - | *cit* | - | - |
| **EcRS58**** | Poultry litter | 6 | 3° | B1 | ENR – NOR – CIP – CHL – SXT – TET – NAL | *-* | I1 – FIB | *bla*_CTX-M 8_ | - | - |
| **EcRS59**** | Water | 4 | 2° | A | ENR – NOR – CIP – TET – NAL | *-* | - | - | - | - |
| **EcRS60**** | Poultry litter | 8 | 2° | A | ATM – FEP – CTX – SXT – TET – NAL | *+* | I1 – FIB | *bla*_CTX-M 2_ | + | - |

(+) Presence; (-) Absence**. *PG**: phylogenetic group; *******E. coli* strains isolated in MacConkey agar without supplement with cefotaxime. **^a^**ESBL phenotype non detected – Failure to detect the ESBL phenotype is related to possible interference from AmpC production in the test.
